# Down-regulating cyclin-dependent kinase 9 of alloreactive CD4^+^ T cells prolongs allograft survival

**DOI:** 10.18632/oncotarget.8804

**Published:** 2016-04-18

**Authors:** Yang Zhan, Yeming Han, Hukui Sun, Ting Liang, Chao Zhang, Jing Song, Guihua Hou

**Affiliations:** ^1^ Laboratory of Experimental Teratology, Ministry of Education and Institute of Experimental Nuclear Medicine, School of Medicine, Shandong University, Jinan, Shandong, China; ^2^ Key Laboratory of Cardiovascular Remodeling and Function Research, Chinese Ministry of Education and Chinese Ministry of Health, Department of Cardiology, Qilu Hospital, Shandong University, Jinan, Shandong, China

**Keywords:** CDK9, cytokines, allorejection, tolerance, CD4^+^ T cell, Immunology and Microbiology Section, Immune response, Immunity

## Abstract

CDK9 (Cyclin-dependent kinase 9)/Cyclin T1/RNA polymerase II pathway has been demonstrated to promote the development of several inflammatory diseases, such as arthritis or atherosclerosis, however, its roles in allotransplantation rejection have not been addressed. Here, we found that CDK9/Cyclin T1 were apparently up-regulated in the allogeneic group, which was positively correlated with allograft damage. CDK9 was inhibited obviously in naive splenic CD4^+^ T cells treated 6 h with 3 μM PHA767491 (a CDK9 inhibitor), and adoptive transfer of these CD4^+^ T cells into allografted SCID mice resulted in prolonged survival compared with the group without PHA767491 pretreated. Decelerated rejection was correlated with enhanced IL-4 and IL-10 production and with decreased IFN-γ production by alloreactive T cells. More interestingly, we found that CDK9_42_, not CDK9_55_, was high expressed in allorejection group, which could be prominently dampened with PHA767491 treatment. The expression of CDK9_42_ was consistent with its downstream molecule RNA polymerase II. Altogether, our findings revealed the crucial role of CDK9/Cyclin T1/Pol II pathway in promoting allorejection at multiple levels and may provide a new approach for transplantation tolerance induction through targeting CDK9.

## INTRODUCTION

The main goal of transplantation immunology research is to induce donor-specific tolerance and to decrease the lifelong need of anti-rejection drugs. Allorejection is mainly mediated by alloreactive CD4^+^ and CD8^+^ T cells, and CD4^+^ T cells have emerged as the most critical factor that may induce allograft destruction in the absence of CD8^+^ T cells, and depletion of peripheral CD4^+^ T cells could apparently prolong allograft survival [1-4]. The current anti-rejection drugs using in clinic, including mTOR and Stat/Jak inhibitors, target only a few genes activated by inflammatory signaling and whose efficacy is limited to particular pathways [5, 6]. Therefore, a strategy that effectively suppresses de novo inflammation by targeting many more primary response genes in allografts may prevent or delay the onset of allorejection.

CDK9, a component of the positive transcription elongation factor b (P-TEFb) kinase, regulates the transcriptional activity of primary response genes by phosphorylating the RNA polymerase II C-terminal domain upon exposure to certain stimuli [7, 8]. Accumulating evidences support a crucial role for CDK9 in monitoring the activation of primary inflammatory response genes and controlling inflammatory processes [9-13]. The differential distribution and regulation of the two isoforms of CDK9, CDK9_42_ and CDK9_55_, may allow greater control of P-TEFb activities under varying cellular states [14-16]. Furthermore, it was reported that CDK9_42_ was localized diffusely in the nucleoplasm, while the CDK9_55_ accumulated in the nucleolus [17].

Thus, this study aimed to investigate whether PHA767491 (a novel selective CDK9 inhibitor) could prolong survival of allografts through reducing the activity of donor-reactive CD4^+^ T cells and which isoform of CDK9 predominantly acts in CD4^+^ T cells activation under allorejection.

## RESULTS

### CDK9 up-regulates allorejection

**Figure 1:**
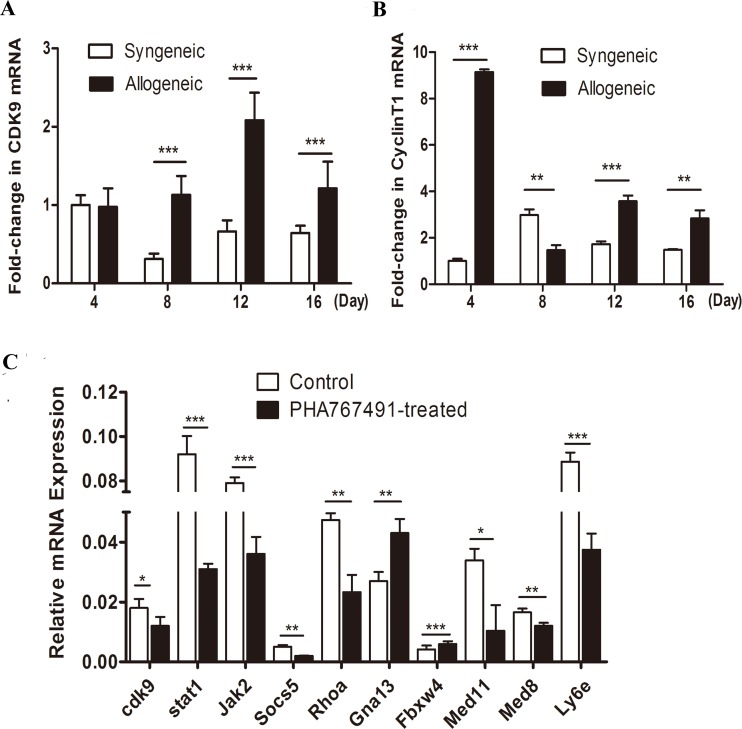
CDK9 is positively associated with the severity of allorejection **A.**-**B.** CDK9 and Cyclin T1 mRNA expression enhanced in allogeneic recipients. The results of Q-RT-PCR assays plotting the fold changes in CDK9 (A) and Cyclin T1 (B) in syngeneic day 4 skin grafts normalized to GAPDH are shown. **C.** CDK9 regulates rejection-related genes in alloreactive CD4^+^ T cells. Cells were isolated from allogeneic transplant recipients at day 12 and then treated with 3 μM PHA767491 or PBS for 2 h followed by assay of reporter gene expression assay. The data are presented as the means±s.d. from three independent experiments. The data are analyzed by Student's t test. *p < 0.05, **p < 0.01, ***p < 0.001.

To investigate the expression of CDK9 during the process of allorejection, we established both syngeneic and allogeneic mouse skin transplant models. Skin grafts were collected on days 4, 8, 12 and 16 after transplantation, and CDK9 and Cyclin T1 mRNA expression levels were quantified. As shown in Figure 1A, CDK9 mRNA significantly up-regulated at the peak rejection period (day 12). As the rejection remitted (day 16), CDK9 expression decreased (Figure 1A). The levels of Cyclin T1 were similar to those of CDK9 (Figure 1B). The expression of CDK9 and Cyclin T1 levels were both dramatically higher in the allogeneic group than in the syngeneic group from day 8 to day 16 (Figure 1A, 1B), especially at day 12, suggesting that CDK9 expression positively correlated with the severity of allorejection.

To further understands the functions of CDK9 in allorejetion, we validated some genes from a CD4^+^ T cell-mediated allorejection SAGE (serial analysis of gene expression) library previously published by our lab [18]. Splenic CD4^+^ T cells were isolated from allogeneic transplant mice on day 12, and then treated with 3 μM PHA767491 (dose-effect of PHA767491 was shown in Supplementary Figure 2) for 2h. As shown in Figure 1C, CDK9 inhibition resulted in dramatic down-regulation in *Stat1* (by 66.3%), *Jak2* (by 54.4%), *Socs5* (by 60%), *RhoA* (by 50.8%), *Ly6e* (by 57.8%), *Med8* (by 27.3%), and *Med11* (by 69.5%) expression, as well as significant up-regulation of *Gna13* (by 59.3%) and *Fbxw4* (by 43.9%) expression. These genes contribute to allorejection, and are mainly involved in transcription (*Med8* and *Med11*), activation (*Ly6e*), proliferation (*Stat1/Jak2* and Socs5), adhesion and movement (RhoA, Gna13, and *Fbxw4*) [19-25]. Our results suggested that CDK9 played a prominent and comprehensive role in promoting the occurrence and development of allorejection at multiple levels.

### CDK9 inhibition of CD4^+^ T cells prolongs allograft survival

**Figure 2:**
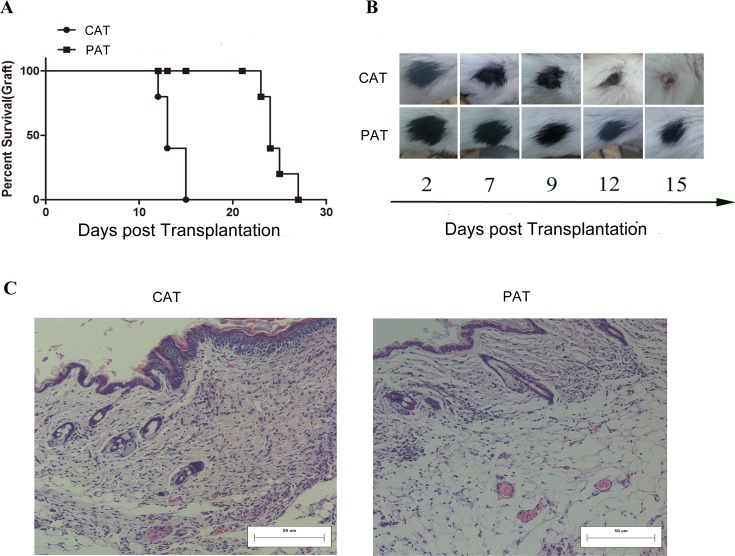
Adoptive transfer of PHA767491-pretreated CD4^+^ T cells prolongs skin allograft survival **A.** SCID allogeneic skin transplant recipients received 1×10^7^ PHA767491-pretreated CD4^+^ T cells (PAT group) at three weeks post-transplantation. Control mice underwent adoptive transfer of PBS-pretreated CD4^+^ T(CAT group) at three weeks post-transplantation. (*n* = 5). **B.** Survival times and conditions of skin allografts. The data are representative of three independent experiments. C. HE staining of skin allografts sections.

To investigate the effect of CDK9 inhibition of CD4^+^ T cells in prolonging allograft survival, SCID allogeneic skin transplantation models were constructed, and naive CD4^+^ T cells pretreated with PHA767491 (PAT) or PBS (CAT) were adoptively transferred. As shown in Figure 2A and 2B, allorejection occurred in the PAT group on days 23~27, whereas on days 12~15 in CAT group. Approximately 46% of the recipients in the PAT group achieved long-term survival (*p* = 0.014). Graft histological assay showed obviously inflammatory infiltration and tissue necrosis in the CAT group while an apparent decrease of inflammation and necrosis was observed in the PAT group (Figure 2C). In addition, the toxicity and the optimal concentration of PHA767491 *in vivo* (3 mg/kg) were determined. Consistent with the adoptive transfer experiment, this dose of PHA767491 *in vivo* administration achieved prolonged graft survival in BALB/c allograft model (Supplementary Figure 1).

### Donor-reactive CD4^+^ T cell response is weakened in PAT mice

**Figure 3:**
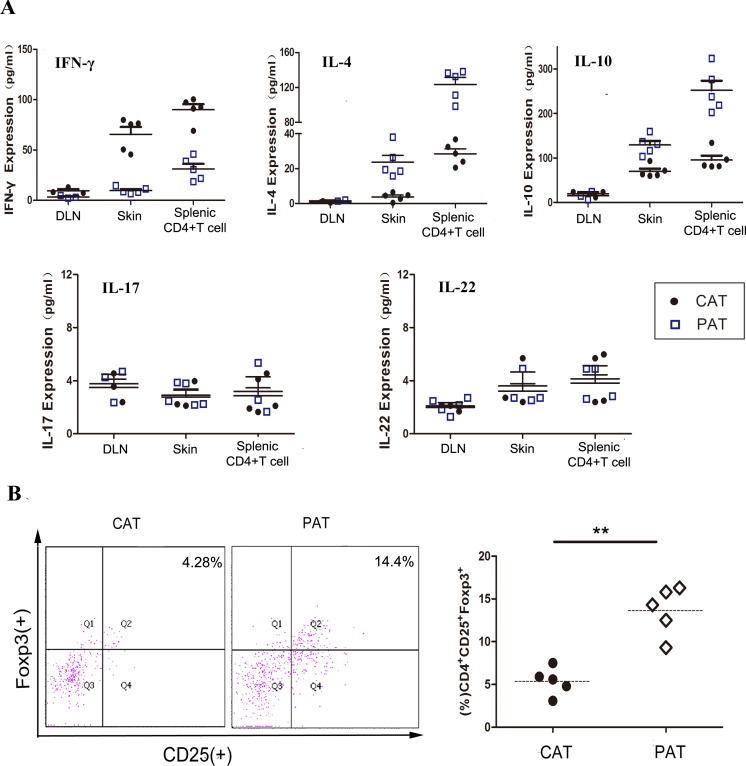
PAT recipients exhibit decreased anti-donor immunity and graft inflammation Analysis was performed on day 12 post adoptive transfer. **A.** The expression of IFN-γ, IL-4, IL-10, IL-17, and IL-22 in cells and tissues was analyzed by multiple cytokines array. Each data point represents an individual mouse. (*n* = 5). **p < 0.01, for unpaired t test between CAT and PAT mouse. **B.** Splenocytes frequencies of Treg were determined by flow cytometry. (*n* = 5).

To further understand the mechanism of CDK9 inhibition prolonged allograft survival, the CD4^+^ T cells adoptive transferred allorecipients were sacrificed on day 12 (acute rejection of the CAT group). Skin graft, CD4^+^ T cells from spleen and draining lymph node were analyzed by antibody array and flow cytometry. PHA767491 treatment apparently suppressed the expression of Th1-type cytokine IFN-γ by 8.2-fold in allografts, by 6.7-fold in splenic CD4^+^ T cells, and obviously increased Th2-type cytokines IL-4 and IL-10 by 4.8 and 1.9 fold in allografts, and by 4.3-fold and 2.6-fold in splenic CD4^+^ T cells, respectively (Figure 3A), while no obvious change was detected in draining lymph node. And also, no significant changes in IL-17 and IL-22 expression were detected in graft, splenic CD4^+^ T cells or draining lymph node (Figure 3A). In addition, we observed a marked increase of splenic Tregs (regulatory T cells) in the PAT group (Figure 3B). These results indicated that CDK9 inhibition may weaken the anti-donor response by a predominance of Th2 and Tregs, and independent of Th17 and Th22 cells.

**Figure 4:**
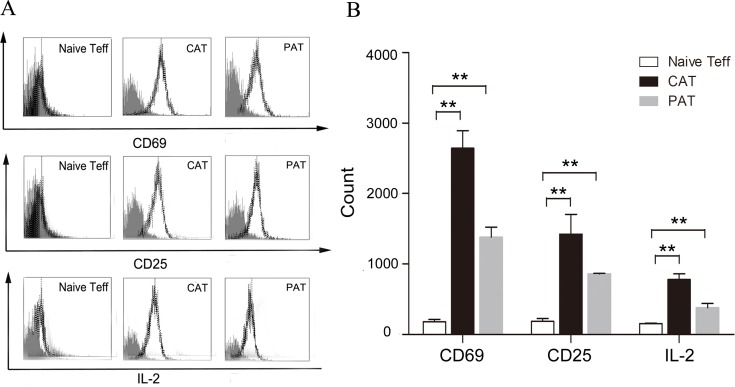
PHA767491 inhibits the activation of alloreactive Teffs **A.** Representative fluorescence histograms demonstrate decreased expression of CD69 and CD25 and intracellular expression of IL-2 in PAT splenic Teffs. Normal lines: isotype-match negative control; black lines: experimental group. **B.** Geometric mean fluorescence intensity ratio ± SEM shows alternative expression. (3-5 mice per group). **p < 0.01.

To investigate whether CDK9 promotes allorejection in the activation stage, we isolated the recipients' splenic Teffs (effector T cells) at day 7 post transplantation, and we found that PHA767491 suppressed the activation of alloreactive Teffs. The expression of CD69, CD25 and IL-2 was decreased by 47.3%, 22.9% or 44.9% in the PAT group, respectively (Figure 4A, 4B). PHA767491 suppressed the activation of alloreactive Teffs.

### PHA767491 decreases Teffs proliferation without change of Tregs proliferative and suppressive capacities

**Figure 5:**
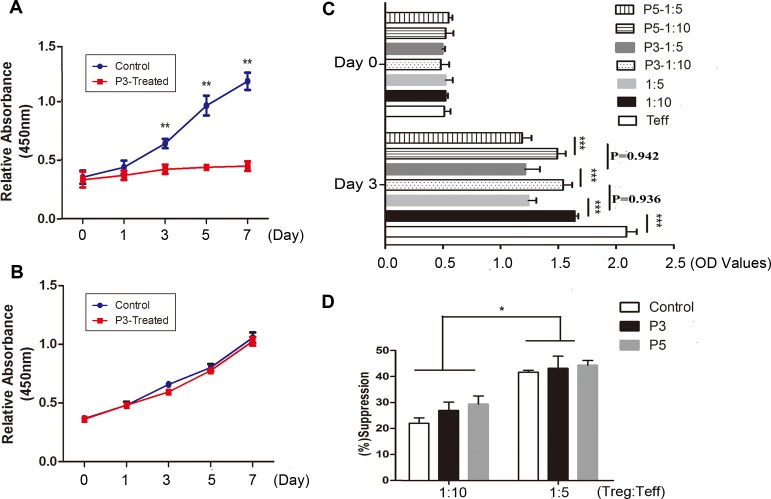
PHA767491 decreases Teffs proliferation without change of Tregs proliferative and suppressive capacity **A.**-**B.** Proliferation of Teffs (A) and Tregs (B) which were incubated for 7 days in the presence of 2 μg/ml anti-CD3 mAb and 1 μg/ml anti-CD28 mAb with 3 μM PHA767491 or PBS. **C.**-**D.** A suppression assay was performed in which Teffs were co-cultured with PHA767491 (3 μM or 5 μM) or PBS-pretreated Tregs at a ratio of 5 to 1 or 10 to 1. Representative proliferation (C) and quantitative suppressive capacities (D) are shown. *p < 0.05, **p < 0.01, ***p < 0.001.

We also detected the effects of CDK9 inhibition on regulatory T cells and Teffs proliferation at day 0, 1, 3, 5 and 7 with anti-CD3/anti-CD28 mAb stimulation. As expected, Teffs expanded robustly in the control group and poorly in 3 μM PHA767491-treated group (Figure 5A). Overall, Tregs expansion in 3 μM PHA767491-treated group showed no difference compared with control group, and the proliferation of two groups considerably increased by 2.8-fold and 2.9-fold respectively on day 7 compared to that on day 1 (Figure 5B).

The effects of PHA767491 on the regulatory activities of Tregs were analyzed after treatment with 3 μM or 5 μM PHA767491.The proliferation of Teffs stimulated by anti-CD3/anti-CD28 mAb was significantly lower when the cells were co-cultured with untreated Tregs, and the higher Treg:Teff ratios had stronger inhibition effects on proliferation. However, no obvious change was observed between co-cultured with pretreated-Tregs and untreated control Tregs (Figure 5C, 5D). The results indicated that CDK9 inhibition showed no apparent influence on the suppressive capacity of Tregs.

### PHA767491 shows greater CDK9_42_ inhibition than CDK9_55_ in CD4^+^ T cells

**Figure 6:**
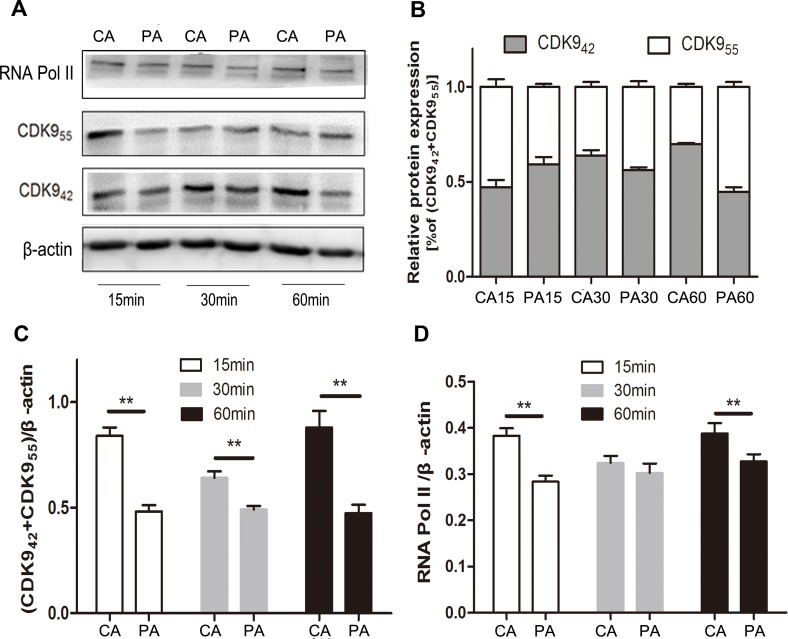
PHA767491 dominantly dampens CDK9_42_ in CD4^+^ T cells **A.** Western blot detection of RNA Pol II, CDK9_55_, CDK9_42_ and β-actin expression in antigen-activated (2 μg/ml anti-CD3 mAb and 1 μg/ml anti-CD28 mAb) CD4^+^ T cells pretreated with 3 μM PHA767491 or PBS for 2 h. **B.**-**D.** The blot and gray value are shown. Similar results were observed in three independent experiments. **p < 0.01.

**Figure 7:**
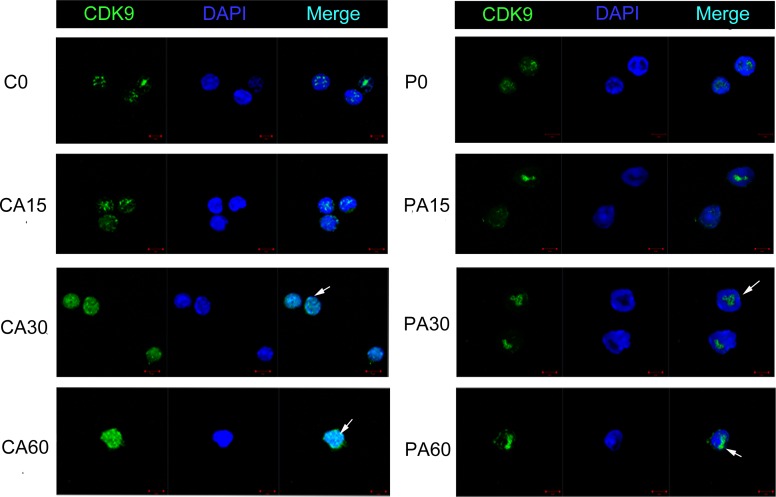
PHA767491 alters the subcellular distribution of CDK9_42_ and CDK9_55_ in CD4^+^ T cells Immunofluorescence staining for changes in the expression of CDK9_42_ and CDK9_55_ in alloantigen-activated CD4^+^ T cells pretreated with PBS or 3 μM PHA767491. Scale bar: 5 mm. Cell nuclei were identified by DAPI staining (*blue*). PHA767491 pretreatment abrogated alloantigen-induced up-regulation of CDK9_42_ expression, but preserved CDK9_55_ expression. All data are representative of three independent experiments (*n* = 4).

In our preliminary experiments, we found that CDK9_42_ was more sensitive to 3 μM PHA767491 treatment than CDK9_55_, total CDK9 (CDK9_42+55_) levels decreased by 54.2%, while CDK9_42_ levels reduced by 66.6% and CDK9_55_ levels reduced by 37.9% (Supplementary Figure 2). We next examined the expression and localization of these two isoforms in alloantigen-stimulated CD4^+^ T cells pretreated with or without PHA767491 for 2 h. Along with the stimulation of alloantigens, CDK9_42_/CDK9_(42+55)_ were increased by 35.1% at 30 min and by 48% at 60 min in the control group. While CDK9_42_/CDK9_(42+55)_ was persistently decreased in the PHA767491 treated group from 15 to 60min (Figure 6B). RNA Pol II expression was consistent with CDK9_42_ expression (Figure 6B, 6D), but not consistent with CDK9_55_ expression. Immunofluorescence assay confirmed this result. The subcellular localization of CDK9 expression gradually moved from the nucleolus (CDK9_55_) to nuclear speckles (CDK9_42_) in the control group, while PHA767491 attenuated this translocation of CDK9_42_ (Figure 7). These data indicate that CDK9_42_, not CDK9_55_, has the dominant role in allorejection.

## DISCUSSION

The key findings of this study include: (1) CDK9 is positively associated with the severity of allorejection, (2) CDK9 promotes activation and proliferation in alloreactive CD4^+^ T cells, and (3) CDK9_42_, not CDK9_55_, enhances alloresponse.

A variety of inflammatory cells and molecules, including neutrophils, lymphocytes, vascular endothelial cells, complement and cytokines, participate in the development of allorejection [26]. Effectively limiting the development of inflammation will greatly delay allorejection. Evidence indicates that CDK9 is the primary target of flavopiridol responsible for its profound anti-inflammatory effects *in vitro* [10, 11]. CDK9 is involved in a central mechanism that activates primary inflammatory response gene transcription in human chondrocytes [7]. CDK9 inhibition has previously been shown to block the CDK9-activated NF-κB pathway to induce strong anti-inflammatory effects in human endothelial cells [27-30]. IL-6 induces CDK9 to bind to Stat3 in the nucleus to abrogate the suppression of p21 by DRB [31, 32].

In our previous work, we identified differentially expressed genes in mouse alloreactive CD4^+^ T cells using the SAGE method [18]. Because CD4^+^ T cells are principal contributors to rejection, we selected several genes from this SAGE library and found that CDK9 promoted transcription, cellular activation and proliferation *via* these genes during CD4^+^ T cell-mediated mouse skin allorejection.

Consistent with prolonged survival of allograft, the inflammatory responses mediated by Th1 cells in the graft site were alleviated in PAT mice, and PHA767491 induced Th2 and Treg differentiation. Significant difference in Th17 was not detected in our experiment which may be due to that it mainly participates in the recruitment of neutrophils [33, 34]. Here, we focused on the allorejection, which is mainly mediated by Th1/Th2 cells. Additionally, Th22 cells have been shown to possibly contribute to skin transplant rejection [35, 36]. However, the effect of Th22 cells was not detected in our study of allorejection promoting function of CDK9.

It was reported that Tregs could suppress the inflammatory response and alleviate rejection through inhibiting on Teffs. And most of immunosuppressants primarily affect Tregs. However, our data indicated that CDK9 inhibition prolonged the allorejection response without apparent impact on Tregs. We also found that the suppressive effect of PHA767491 was much stronger on Teffs than Tregs.

CDK9 is known as an effective target for eliciting apoptosis [37], so we investigate whether PHA767491 suppresses Teff activity *via* an apoptotic pathway. We found that PHA767491 promoted early apoptosis with allo-antigen stimulation and no effect upon the cell cycle (Supplementary Figure 3).

The CDK9_42_ isoform has been shown to play a more important role than CDK9_55_ in responding to a variety of stimuli. Therefore, we investigated the effect of PHA767491 on isoforms CDK9_55_ and CDK9_42_ respectively. Consistent with a previous report [14], the ratio of CDK9_55_ to CDK9_42_ significantly decreased in activated CD4^+^ T cells, however, PHA767491 treatment reversed this pattern, inhibiting CDK9_42_ expression without disturbing CDK9_55_ expression. These data indicated that CDK9_42_, not CDK9_55_, played a predominant role in responding to stimulation and downstream pathways activating. Since CDK9_55_ has been reported to maintain the essential function of CDK9, this effect may avoid the loss of essential biological functions of CDK9 under special conditions.

Our data demonstrate that the CDK9/Cyclin T1/RNA Pol II pathway plays a central role in the development of allorejection in mice models. The CDK9 inhibitor suppresses transcription of allorejection-related response genes, leading to the inhibition of alloreactive Teff activation and proliferation and the weakened alloresponse through a predominance of Th2 and Tregs and independent of Th17 and Th22 cells. More importantly, we showed that CDK9_42_, not CDK9_55_, played a dominant role during allorejection. Our finding provided a new insight into the mechanism underlying allograft tolerance and a new approach for prolonging allografts through targeting CDK9.

## MATERIALS AND METHODS

### Animals

Female BALB/c (H-2d) and C57BL/6 (H-2b) mice weighing 18±2 g were purchased from the Laboratory Animal Center, Shandong University. Female SCID (H-2d) mice weighing 18±2 g were purchased from the Institute of Laboratory Animal Sciences (Beijing, China). SCID mice were given free access to standard mouse chow and tap water. All mice were treated in strict compliance with protocols approved by the Institutional Animal Care and Use Committee (IACUC). Experiments were performed in accordance with national animal protection guidelines.

### Allogeneic and syngeneic skin transplant models

To establish an allogeneic transplantation model, full-thickness skin grafts from donor C57BL/6 mice were transplanted onto prepared graft beds in the right shoulders of recipient BALB/c mice. To establish a syngeneic transplantation model, full-thickness skin grafts from donor BALB/c mice were transplanted onto prepared graft beds in the right shoulders of recipient BALB/c mice. Transplantations were performed as previously described [38]. In a separate experiment, mice were treated with normal saline or PHA767491 (3, 10 or 30 mg/kg) by oral administration on day 1, 3, 5, 7, 10 and 14 post transplantation. Grafts were observed daily after bandages were removed from recipients at day 7; rejection was defined as necrosis of more than 90% of the graft.

### SCID allogeneic skin transplant model

Transplantation was performed as previously described [38]. Briefly, full-thickness skin grafts from donor C57BL/6 mice were transplanted on BALB/c SCID recipients, and the grafts were allowed to heal for approximately 2-3 weeks.

### Cell preparation and adoptive transfer

Single spleen cells suspension of BALB/c mice were prepared according to standard procedures. CD4^+^ T cells were enriched using an EasySep™ Mouse CD4^+^ T Cell Enrichment Kit (Stemcell Technologies, Vancouver, BC Canada) according to the manufacturer's instructions. Tregs were isolated using an EasySep™ Mouse CD4^+^CD25^+^ Regulatory T Cell Isolation Kit (Stemcell Technologies, Vancouver, BC Canada). The magnetically labeled CD4^+^CD25^+^ T cells were remained inside the tube, and CD4^+^CD25^neg^ T cells (Teffs) in the supernatant were purified for use.

For the adoptive transfer experiment, 1×10^7^ CD4^+^ T cells from naive BALB/c mice were treated with 3 μM PHA767491 (PAT group) or with the same volume of PBS (CAT group) for 6 h. The cells were washed twice with PBS and then injected intravenously into allo-SCID-recipients. The graft was observed daily after 7 days, and rejection was defined as graft necrosis of more than 90%.

### Quantitative real-time PCR

Total RNA (2.5 mg) was reverse transcribed into cDNA using a FastQuant RT Kit (TIANGEN BIOTECH, Beijing, China). Quantitative-PCR (qPCR) was performed using SuperMix (Platinum SYBR Green qPCR Kit; CWBIO, Shanghai, China) and a PikoReal 96 system (Thermo Scientific, Schwerte, Germany) as previously described [39]. The specific primer sequences used for CDK9, CyclinT1, Ly6e, Rhoa, Med8, Med11, Socs5, Fbxw4 and Gna13 are included in Table S1.

### Antibody array assay of multiple cytokines

Skin grafts, splenic CD4^+^ T cells and draining lymph nodes were harvested from PAT or CAT mice at the day 12 post adoptive transfer. The cells and tissue lysates were prepared and analyzed using a multiple cytokine array (Suzhou SJ Biomaterials, Jiangsu, China). The arrays were imaged using an ECL system.

### Flow cytometry analysis

To detect the frequency of Tregs, the following fluorescence-conjugated monoclonal antibodies and isotype controls were used: anti-CD69 (H1.2F3), anti-CD25 (PC61.5), and anti-CD4 (GK1.5) (eBioscience, San Diego, CA, USA). Flow cytometry was performed using a BD FACS Calibur (BD Biosciences, San Jose, CA, USA). The data were analyzed using FCS Express V3 (De Novo Software, Los Angeles, CA, USA).

### Intracellular cytokine staining

Detection of intracellular IL-2 in CD4^+^ T cells was performed as previously described [40]. For intracellular Foxp3 staining, cells were fixed and permeabilized using Fix and Perm reagents (Biolegend, San Diego, CA, USA), respectively, and then washed in wash buffer and stained using a PE-Cy5 anti-mouse Foxp3 staining kit (eBioscience) according to the manufacturer's instructions.

### *In vitro* proliferation assay

Teffs or Tregs were seeded at 1×10^4^ cells per well in 96-well plates. After the cells were treated with or without 3 μM PHA767491 in the presence or absence of anti-CD3 mAb (2 μg/ml) and anti-CD28 mAb (1 μg/ml) for 24 h, cell proliferation was measured by CCK-8 assay (Beyotime, Shanghai, China) at 0, 24, 72, 120 and 168 h according to the manufacturer's instructions. Absorbance was measured at a wavelength of 450 nm, OD. Triplicate-repeated wells for each group were measured.

### *In vitro* suppression assay

Purified Teffs (1×10^5^) were cultured with Tregs pretreated with 3 μM or 5 μM PHA767491 or without CDK9 inhibitor in the presence of anti-CD3 mAb (2 μg/ml) and anti-CD28 mAb (1 μg/ml) for 72 h. Cell proliferation was assessed using a CCK-8 proliferation kit, according to the manufacturer's instructions.

### Western blot analysis

Western blot analysis was performed as previously described [41]. Briefly, proteins were extracted from spleen or splenic CD4^+^ T cells from naive BALB/c mice using lysis buffer, and the lysates were boiled for 5 min. The samples were then separated using SDS-PAGE and transferred by electroblotting to polyvinylidene fluoride (PVDF) membranes. The membranes were probed with primary antibodies directed against CDK9 (Cell Signaling Technology, Danvers, MA, USA) or against the internal control β-actin (Santa Cruz Biotechnology, Santa Cruz, CA). The blots were then probed with horseradish peroxidase (HRP)-conjugated secondary antibodies (Santa Cruz Biotechnology), and the chemiluminescent signals were visualized using an ECL-Plus system (Millipore, Billerica, MA, USA). Signals were quantified using a GelDoc XR+ system (Bio-Rad, California, USA).

### Immunofluorescence

Splenic CD4^+^ T cells were fixed in 4% paraformaldehyde and permeabilized using 0.1% Triton X-100. The cells were subsequently stained with an anti-CDK9 antibody, and DAPI was used to stain the nuclei. The cells were then probed with Alexa Fluor 488-conjugated anti-mouse IgG (Molecular Probes). To study the effect of PHA767491 on CDK9 translocation, the cells were treated with 3 μM PHA767491 or left untreated for 6 h and then stimulated with anti-CD3 mAb (2 μg/ml) and anti-CD28 mAb (1 μg/ml) for 15, 30 or 60 min. The cells were fixed and then observed under a LSM 780 fluorescence microscope (Carl Zeiss AG, Cologne, Germany); images were captured using ZEN software.

### Statistical analysis

Two-tailed t-tests were used in comparisons of two groups to evaluate differences. Survival analyses between groups were performed using the log-rank method. All results were generated using GraphPad Prism 5 software (San Diego, CA). *P* values < 0.05 were considered significant. Error bars reflect the standard error of the mean.

## SUPPLEMENTARY MATERIAL FIGURES AND TABLE


