# Supplementary material for: Down-regulating cyclin-dependent kinase 9 of alloreactive CD4+ T cells prolongs allograft survival
Source: Oncotarget. 2016 Apr 18;7(18):24983–94. doi: 10.18632/oncotarget.8804 (PMC5041884; doi:10.18632/oncotarget.8804)
Supplement: Supplementary file 1 [file oncotarget-07-24983-s001.pdf]

Down-regulating cyclin-dependent kinase 9 of alloreactive $CD4^+$ T cells prolongs allograft survival

Supplementary Material

MATERIALS AND METHODS

Alloantigen Preparation

1×10^7 C57BL/6 mouse splenocytes were prepared in 1ml PBS, and then cells were broken by ultrasound (10 seconds, 10 times). Centrifuged, and the supernatant was collected and used as soluable alloantigen. In western blot, apoptosis and cell cycle experiment, control group and P3-treated group were activated by 200 μl alloantigen, and then the latter were treated with 3μM PHA767491 for 2 h.

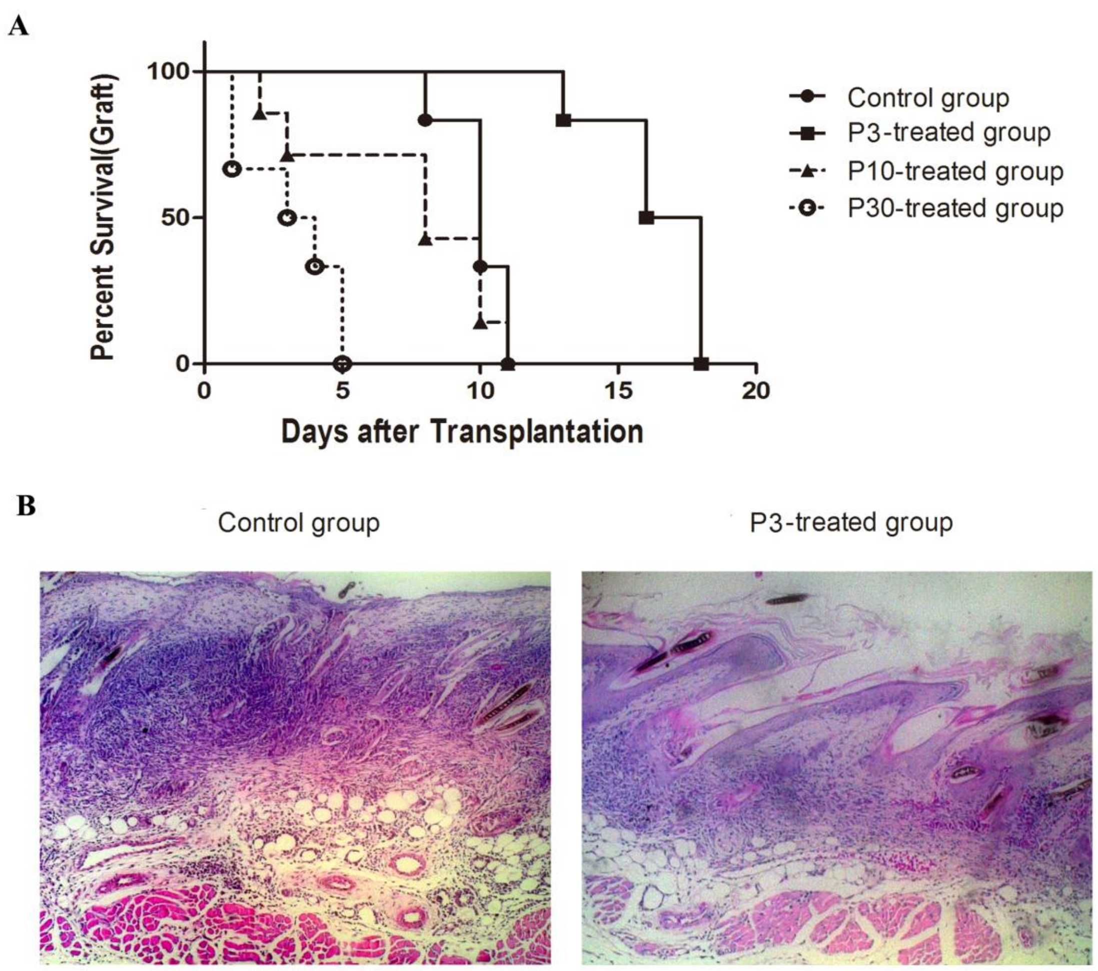

Suppl. Figure 1. **A.** Survival of allotransplanted BALB/c mice treated with PHA767491 *in vivo*. Recipients received either vehicle control (WT; *n*=6) or PHA767491 (3, 10, 30 mg/kg) 1 day before transplantation (P3-treated group: log-rank $p<0.001$; *n*=6). **B.** HE staining of skin allografts .

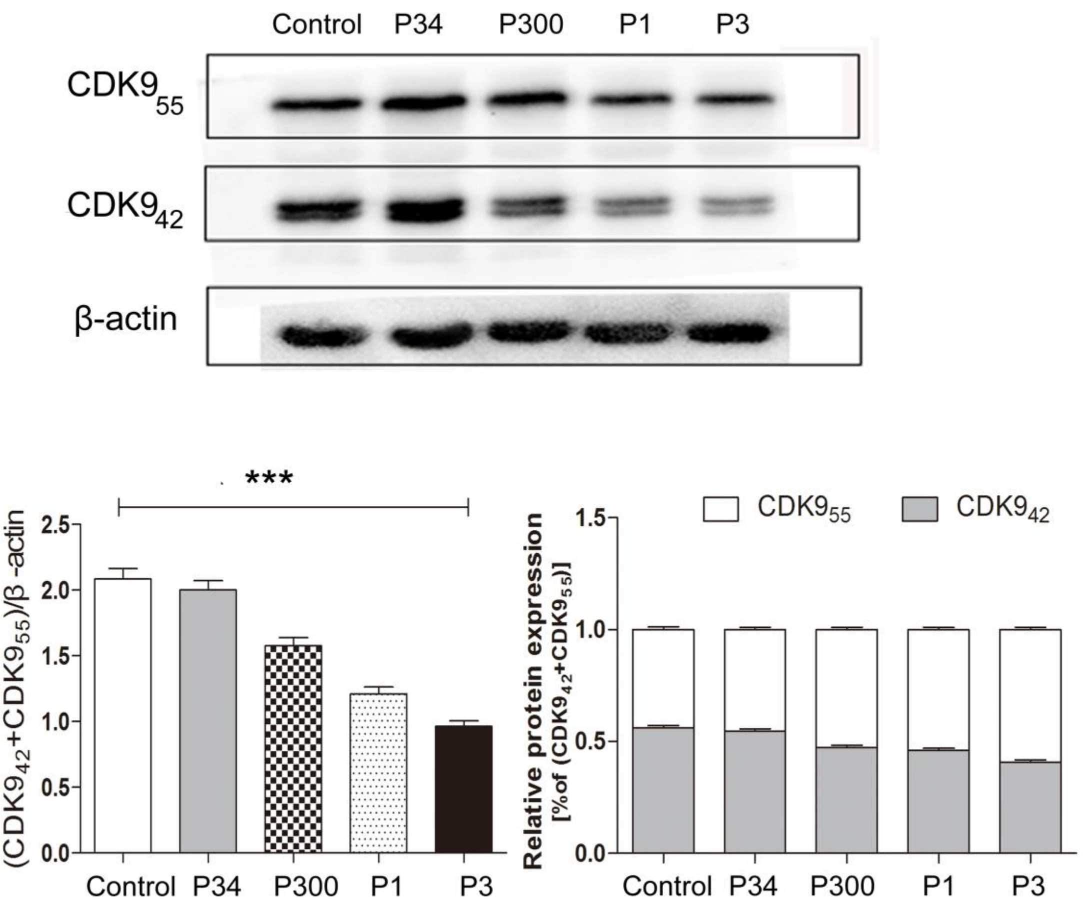

Suppl. Figure 2. Determination of the optimal drug concentration. Western blot of CDK9$_{42}$, CDK9$_{55}$ and β-actin in splenocytes treated with 34 nM, 300 nM, 1 μM and 3 μM PHA767491 or with the same volume of PBS for 2 h. The ratio of CDK9/ β-actin gray value showed that the level of both CDK9$_{42}$ and CDK9$_{55}$ significantly decreased at 3 μM concentration. ***$p<0.001$.

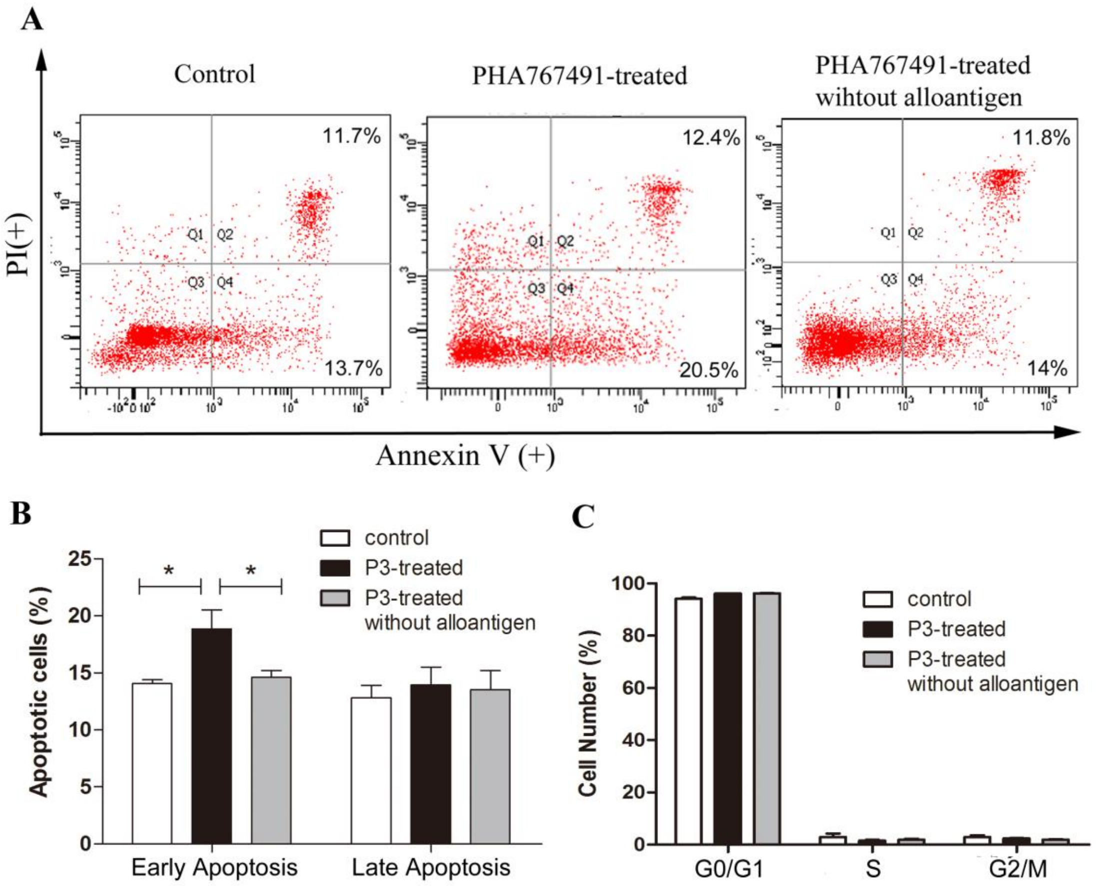

Suppl. Figure 3. PHA767491 promote apoptosis of CD4$^+$ T cells *in vitro*. After treated with 3μM PHA767491 for 2 h, apoptosis and cell cycle were measured by flow cytometry. **A.** The flow cytometry dot plots indicate the frequency of Annexin V$^+$ /PI$^-$. **B.** The bar indicate the summarized data of A (mean ± s.d. compared with CAT). The data are representative of three independent experiments. **C.** The cell cycle is shown in a bar graph form with the G0/G1, S and G2/M phases. PI, propidium iodide.

Suppl. Table 1. Primers used during the study.

Primer name	Sequence
CDK9-F	AGGTCTCCCTGCTTCCTCCT
CDK9-R	AGATGCTGACGGATGGTTTC
Cyclin T1-F	TGCCATCTGCTAAGGTTTCAC
Cyclin T1-R	GACTTCACATTGGCCTCCAT
Ly6e-F	CTTCACCCGGAGTAGGCTTC
Ly6e-R	ATGAACTTGCTCCATGCCCA
Rhoa-F	CAGCAAAGACCAAAGATGGAG
Rhoa-R	ACAAGATGAGGCACCCAGAC
Med8-F	CTGGATTGAAAGTGGGAAGG
Med8-R	AGAACGCTGAGAAAGGCAAC
Med11-F	GCTCAAATCCGCTACCTCAC
Med11-R	GGTTCTCGCCACATCACTAA
Socs5-F	CCGCTACAACAGGTCTCTGC
Socs5-R	CGTGACAGTGGAGGAGTGAA
Fbxw4-F	CAGGAGGTGAACTGTGTGGA
Fbxw4-R	TCGGTCTGGATGGTGTGTAA
Gna13-F	AAGGCAGCAGAAGTCAGTCC
Gna13-R	CGGTCACAATGGAGGAAGTC
